# *In Vitro * Synergistic Antioxidant Activity and Identification of Antioxidant Components from *Astragalus membranaceus* and *Paeonia lactiflora*

**DOI:** 10.1371/journal.pone.0096780

**Published:** 2014-05-09

**Authors:** Xiaoyan Xu, Feng Li, Xin Zhang, Pengcheng Li, Xing Zhang, Zhaoxi Wu, Dapeng Li

**Affiliations:** 1 Department of Food Science, Shandong Agricultural University, Taian, China; 2 College of Life Science, Shandong Agricultural University, Taian, China; 3 National Research Center for Apple Engineering and Technology, Taian, Shandong, China; University of Lancaster, United Kingdom

## Abstract

Many traditionally used herbs demonstrate significantly better pharmacological effects when used in combination than when used alone. However, the mechanism underlying this synergism is still poorly understood. This study aimed to investigate the synergistic antioxidant activity of *Astragalus membranaceus* (AME) and *Paeonia Lactiflora* (PL), and identify the potential antioxidant components by 1,1-diphenyl-2-picrylhydrazine (DPPH) radical spiking test followed by a high performance liquid chromatography separation combined with diode array detection and tandem mass spectrometry analysis (DPPH-HPLC-DAD-MS/MS). Eight AME-PL combined extracts (E_1_–E_8_) were prepared based on bioactivity-guided fractionation. Among them, E_1_ exhibited the strongest synergistic effect in scavenging DPPH radicals and reducing ferric ions (*P*<0.05). Moreover, E_1_ presented strong cytoprotection against H_2_O_2_-induced oxidative damage in MRC-5 cells by suppressing the decrease of the superoxide dismutase (SOD), glutathione peroxidase (GSH-Px) and catalase (CAT) activities. A strong correlation between the increment of total phenolic/flavonoid and synergistic antioxidant activity, especially between the increment of total flavonoid and the increase in ferric reducing power was observed. Finally, seven antioxidant substances were identified in E_1_ as oxypaeoniflora, catechin, calycosin-7-O-β-D-glucopyranoside, fomononetin-7-O-β-D-glucopyranoside, 9,10-dimethoxy-pterocarpan-3-O-β-D-glucopyranoside, quercetin and 2′-dihydroxy-3′,4′-dimethyl-isoflavan-7-O-β-D-glucopyranoside.

## Introduction

Many medicinal herbs are believed to share a common origin with food in Chinese tradition, thus have frequently been used as functional foods or dietary supplements in the East for centuries. It is evidenced that they generally demonstrate significantly higher health-promoting effects when used in the form of multi-herb formulas than when used alone. However, interactive actions among components in these multi-herbs and the involved mechanism remain poorly understood.

Phenolic substances and flavonoids are increasingly recognized as the major bioactive components contributing to the antioxidant potency of many herbs. For example, Fattahi *et al.* [1] found that the significant antioxidant activity of *Dracocephalum kotschyi* was correlated with the flavonoid content. Misbah *et al.* [2] reported that the antioxidant activities of the fruits of *F. deltoidea* might be asserted by the phenolic content. The combination of *Vernonia amygdalina* and *Azadirachta indica* showed a positive synergism in antioxidant action, due to a boost in the flavonoid content of the extracts [3]. The mechanisms of antioxidant activity of phenolics and flavonoids can be characterized not only by directly scavenging or quenching free radicals, but also by inducing various intracellular antioxidant enzymes [4]. Nagata *et al.* [5] revealed that cytoprotective effect of quercetin and catechin against H_2_O_2_ cytotoxicity in rat hepatocytes BL-9 was related to the activation of GPx. Leung *et al*. [6] provided evidence that luteolin-induced human lung carcinoma CH27 cell apoptosis was accompanied by activation of SOD and CAT. Furthermore, some antioxidant effects may be a reset of a combination of radical scavenging and the interaction with enzyme functions [7]. For instance, ethyl acetate-extracted fraction of *Ficus glomerata,* rich in phenolic compounds, possessed high potency to scavenge reactive oxygen species/free radicals and restore the levels of GSH, SOD and CAT [8]. However, these studies mainly focused on the cytoprotective or antioxidant effects of the phenolic compound, flavonoid or extracts from single herb, and limited information is available regarding interactive actions among them.

*Atractylodes macrocephala* (AME) and *Paeonia lactiflora* (PL) are very popular medicinal herbs in some Asian countries, which are commonly used in combination as dietary supplements. Our preliminary results indicated that AME and PL were able to result in a significant synergy in scavenging the DPPH radical, hydroxyl radical and superoxide radical anions [9]. In this paper, to elucidate the potential mechanism by which AME and PL synergistically exerted antioxidant effects, we performed a DPPH scavenging activity-guided fractionation, and investigated the protective effect of the obtained antioxidant components against H_2_O_2_-induced oxidative damage using a MRC-5 cells model.

## Materials and Methods

### Chemicals

1,1-Diphenyl-2-picrylhydrazine (DPPH), 2,4,6-tripyridyl-s-triazine (TPTZ), Trolox, 3-(4,5-dimethylthiazol-2-yl)-2,5-diphenyl tetrazolium bromide (MTT), dimethyl sulfoxide (DMSO), folin-ciocalteau reagent (FCR), gallic acid, rutin (≥95%) and vitamin E (V_E_) were purchased from Sigma-Aldrich Chemical Co. (St. Louis, MO). Dulbecco's modified Eagle's medium (DMEM), trypsin-EDTA (0.25% trypsin with EDTA-4Na), fetal bovine serum (FBS) and penicillin-streptomycin were from Gibco (Grand Island, NY). All other chemicals were of analytical grade and obtained from Shanghai Chemical Reagent Co. (Shanghai, China).

### Plant Materials

*Astragalus membranaceus* Bge. var. mongholicus (Bge.) Hsiao (AME) and *Paeonia lactiflora Pall* (PL) were commercially purchased from Shijiazhuang Pharmaceutical Group (Shijiazhuang, China). They were stored at room temperature until further use.

### Isolation of Antioxidant Components from AME and PL

Each sample was comminuted and sieved through a No. 40 mesh. One kilogram of powder sample (AME or PL) was Soxhlet-extracted with 4 L of 95% ethanol for 2 h. The extraction was repeated for 3 times. The extracts were combined and evaporated under reduced pressure to obtain dry matter. Then, the residue was re-suspended in 500 mL water (OH) and subjected to further sequential extraction by refluxing with the equivalent volume of petroleum ether (PE), chloroform (CF), ethyl acetate (EA) and n-butanol (NB). For each extract, solvents were removed by vacuum evaporation or lyophilization to obtain dry matter. Finally, ten fractions, namely PE-AME, CF-AME, EA-AME, NB-AME, OH-AME, PE-PL, CF-PL, EA-PL, NB-PL and OH-PL, were obtained with the yields of 0.36%, 0.94%, 7.54%, 12.71%, 78.45%, 0.52%, 2.99%, 10.91%, 25.32%, 60.26%, respectively. Preliminary antioxidant assay revealed that EA-PL fraction had the remarkable synergistic effect in scavenging DPPH radicals when used in combination with AME fractions, and was further subjected to silica gel chromatography by stepwise elution with methanol/chloroform (methanol/chloroform  = 1∶19, 1.5∶18.5, 2∶18, 2.5∶17.5, 3∶17, 4∶16, 5∶15, 20∶0, v/v). The elutions were collected and concentrated to finally afford eight fractions (A_1_–A_8_).

### Determination of the Antioxidant Activities

#### Ferric Reducing Antioxidant Power (FRAP) Assay

The FRAP assay was performed according to the method of Benzie and Strain [10] with some minor modifications. One hundred milliliter of sample was mixed with 3.9 mL FRAP reagent consisting of ferric chloride (20 *µ*M) and TPTZ (10 *µ*M). After 10 min, the absorbance was recorded at 593 nm using a UNICO UV-2000 spectrophotometer (Shanghai Instruments Co. Ltd., Shanghai, China). The reducing power was calculated using the absorbance difference between sample and blank and a further parallel ferrous sulfate standard solution. The results were expressed as micromoles of FeSO_4_ per gram dry weight (*µ*mol Fe^2+^/g DW).

#### DPPH Free Radical Scavenging Assay

The DPPH radical scavenging capacity of the samples was evaluated using the modified method reported earlier [11]. Briefly, 0.1 mL sample was added to 3.9 mL of 0.1 mM DPPH ethanol solution, and allowed to stand for 30 min in the dark at 37°C. Then, the absorbance of the reaction solutions was recorded at 517 nm immediately. Percent inhibition of the DPPH radical by the sample was calculated according to the following equation:
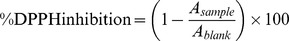
where *A_sample_* is the absorbance of DPPH solution after reacting with a given concentration of sample, and *A_blank_* is the absorbance of DPPH solution with an ethanol blank instead of a sample. The percentage of DPPH reduced was plotted against the concentration of each sample, and an SC_50_ value (the concentration required to scavenge 50% of the DPPH) was calculated.

### Determination of Total Phenolic and Flavonoid Contents

The determination of total phenolic content was carried out by the Folin-Ciocalteau method as reported previously [12]. Briefly, 0.1 mL of sample was mixed with 1 mL of the Folin-Ciocalteau reagent and incubated at room temperature for 5 min. Then, 1 mL of 0.1 g/mL Na_2_CO_3_ solution was added to the mixture. After 90 min incubation, the absorbance of the reaction mixture was recorded at 765 nm. Total phenolic content was calculated by comparison with a standard curve generated by analyzing gallic acid. The results were expressed as gallic acid equivalents (GAE) per gram of sample.

Total flavonoid content of the samples was determined according to colorimetric method as described previously [13]. Briefly, 0.1 mL of sample was mixed with 0.3 mL of 0.05 g/mL NaNO_2_ solution in a test tube and incubated for 5 min. Then, 0.3 mL of 0.1 g/mL AlCl_3_ solution was added and incubated for another 6 min. The reaction was terminated by adding 2 mL of 1 M NaOH solution. The absorbance of the reaction mixture was recorded immediately at 510 nm. Total flavonoid content was calculated by comparison with a standard curve generated from rutin, and the results were expressed as the mg rutin equivalents (RE) per gram of sample.

### Cell Culture

Lung fibroblasts MRC-5 cells, obtained from Shanghai Institute of Cell Biology (Shanghai, China), were cultured in Dulbecco's modified Eagle's medium (DMEM), supplemented with 10% fetal bovine serum (FBS), 100 U/mL penicillin and 100 *µ*g/mL streptomycin at 37°C in a humidified atmosphere of 5% CO_2_.

### Cell Viability Assay

The effect of different fractions on cell viability was evaluated by MTT assay as described previously [14], [15], which was based on the reduction of a tetrazolium salt by mitochondrial dehydrogenases in viable cells. MRC-5 cells were seeded in 96-well plates at a density of 2×10^5^ cells/mL. After 24 h of preconditioning, the cells were subjected to five different treatments, i.e., H_2_O_2_ (0.5 mM), H_2_O_2_+V_E_ (5 *µ*g/mL), H_2_O_2_+CF-AME (7 *µ*g/mL), H_2_O_2_+A_1_ (3 *µ*g/mL), H_2_O_2_+E_1_ (5 *µ*g/mL). The cells were incubated for additional 24 h. Subsequently, 10 *µ*L of 5 mg/mL MTT dye solution was added to each well and the plate was incubated for 4 h at 37°C. Then, 100 *µ*L of DMSO was added to each well and kept for 15 min to dissolve tetrazolium dye. Relative cell viability was calculated by determining the absorbance at 570 nm and untreated control cells were assigned a relative viability of 100%.

### Activity of Cellular Antioxidant Enzymes and Malonaldehyde (MDA) Content

Logarithmically growing MRC-5 cells were split into six culture flasks at a density of 2×10^5^ cells/mL and incubated for 24 h. The medium was replaced with fresh medium containing the following samples, A: control (without H_2_O_2_ or antioxidant fractions), B: H_2_O_2_ (0.5 mM), C: H_2_O_2_+V_E_ (5 *µ*g/mL), D: H_2_O_2_+CF-AME (7 *µ*g/mL), E: H_2_O_2_+A1 (3 *µ*g/mL), F: H_2_O_2_+E1 (5 *µ*g/mL), and the cells were incubated for additional 24 h. After removing the medium, the cells were suspended in 10 mM PBS (pH 7.4) and lysed suing a JY92-IIY ultrasonic cell disruption system (Beidi Experiment Instrument Inc., Nanjing, China). The lysates were centrifuged at 4,000 rpm and 4°C for 10 min. The supernatants were used for measurement of cellular MDA content and antioxidant enzymes (SOD, CAT and GSH-Px) using the commercially available assay kits (Jiancheng Biochemical Inc., Nanjing, China).

### On-line DPPH-HPLC-MS/MS Assay

Identification of the potential antioxidant components in AME-PL extract was performed using a DPPH-HPLC method coupled with an on-line HPLC-DAD-MS/MS technique [16]. Briefly, 400 *µ*L of each sample was mixed with 600 *µ*L of DPPH ethanol solution (0.25 mg/mL) and then reacted for 30 min in the dark at room temperature. The mixture was filtered through a 0.45 *µ*m filter membrane and subjected to HPLC separation. The control sample was prepared by adding 600 *µ*L ethanol instead of DPPH to each sample.

The separation of the antioxidant components was conducted by an Agilent LC-1200 high performance liquid chromatograph (Agilent Inc., Palo Alto, CA) equipped with a XB-C_18_ analytical column (4.6 mm×250 mm, 5 *µ*m) and a photodiode array detector (DAD). HPLC separation was performed using a linear gradient of A (acetonitrile) and B (water) at 30°C at a flow rate of 0.8 mL/min. The solvent gradient was used as follows: 0–10% A (0–10 min), 10–11.2% A (10–15 min), 11.2–15% A (15–25 min), 15–22% A (25–30 min), 22–27.7% A (30–43 min), 27.7–37% A (43–59 min), 37–65% A (59–79 min), 65–72% A (79–84 min), 72–85% A (84–97 min), 85–100% A (97–100 min). The injection volume was 15 *µ*L. Detection wavelength was set at 269 nm and UV spectra from 200 to 600 nm were also recorded for peak identification.

In order to identify the screened antioxidant components, a Waters Micromass Quattro Ultima PT system equipped with an electrospray ionization (ESI) source and a data analysis software MassLynx 4.0 SP4 was used (Waters Corporation, Milford, MA). The flow rate of 0.8 mL/min was reduced to 0.2 mL/min before the eluent entered the ESI interface. The mass spectrometry detector (MSD) parameters were set as follows: positive ion mode; cone voltage 45.0 V; collision energy 20.0 eV; mass analyzer scanned from 80 to 1500 u. The MS/MS spectra were recorded in auto-MS/MS mode.

### Determination of Combination Index

To investigate the possible interaction between different extracts, an isobolographic analysis based on the median-effect principle was performed [17]. The median-effect principle was used to calculate individual and combined AME and PL effects. Dose-effect curves for each fraction from AME or PL and their combinations with series diluted concentrations were plotted by using the median-effect equation:
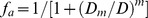
where D is the dose. *D*_m_ is the dose required for 50% inhibition effect, which is equivalent to median-effect dose (EC_50_). 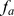 is the fraction affected by dose *D*, and *m* is a coefficient of the sigmoidicity of the dose-effect curve.

The medium-effect plot is based on the logarithmic form of Chow's median-effect equation [18]:
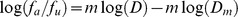
where 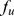 is the fraction unaffected, 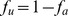.

Combination index (CI) based on the classic isobologram equation is used for data analysis of two-way combination [18]:
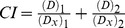



where (D)_1_ and (D)_2_ are the doses of the fractions from AME and PL, respectively, in the combination system; (D_x_)_1_ and (D_x_)_2_ are the doses of the fractions from AME and PL alone, respectively. CI>1, CI = 1, and CI<1 represent antagonism, additivity or synergism, respectively. In order to analyze the interactive effect qualitatively and quantitatively, the two herbs were administered at a fixed mass ratio of 1∶1.

### Statistical Analysis

All experiments were carried out in triplicate and data were expressed as mean ± standard deviations. A one-way analysis of variance (ANOVA) was performed to calculate statistical differences, and multiple comparisons of means were done by the Duncan test using statistical software SPSS 13.0 (SPSS Inc., Chicago, IL). A probability value of <0.05 was considered significant.

## Results and Discussion

### DPPH-scavenging Capacity of the Combined Fractions from AME and PL

**Figure 1:**
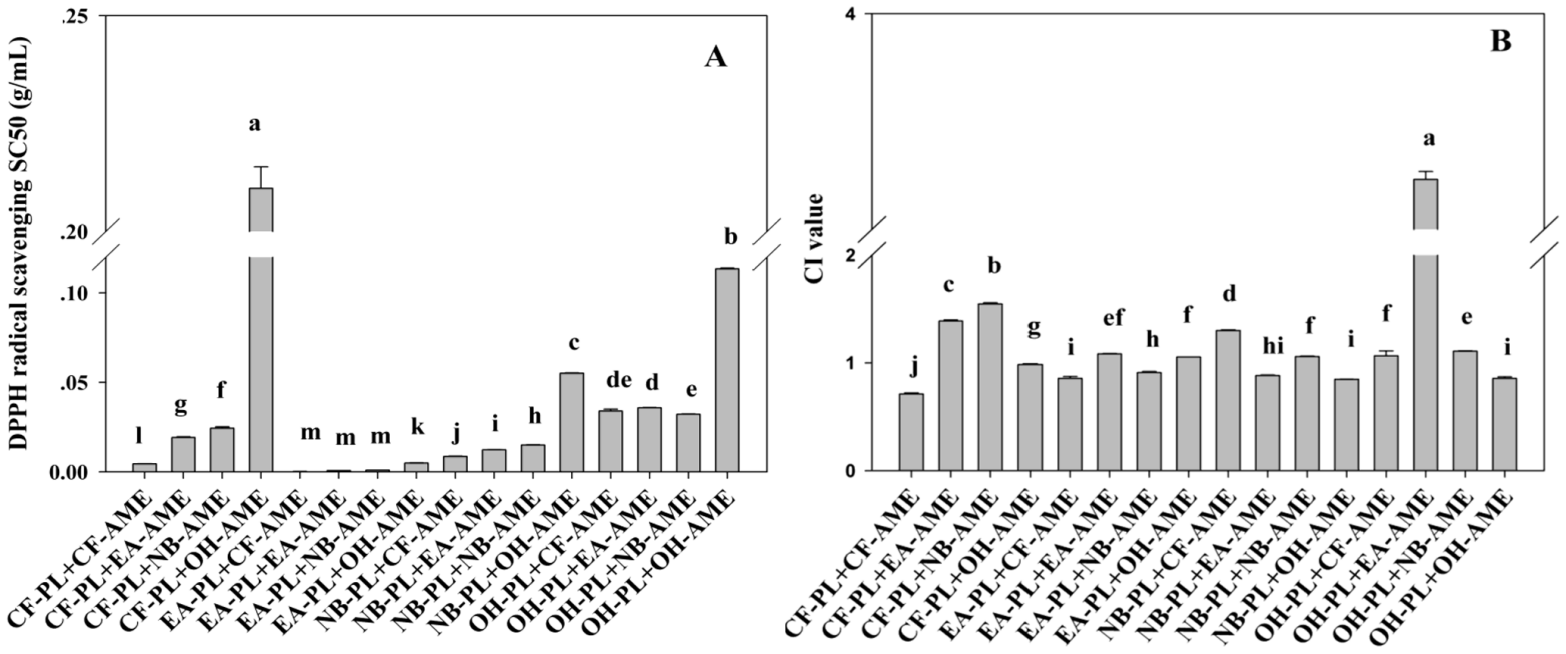
DPPH radical-scavenging activity (A) and CI values (B) of different combinations of AME and PL solvent-extracted fractions. Data are expressed as means ± SD (n = 3), and histograms marked with different letters are significantly different at *P*<0.05.

The DPPH radical-scavenging activity of different combined fractions from AME and PL are shown in Fig.1. Sixteen combined fractions exhibited a wide range of differences in scavenging DPPH free radicals. Among them, the ethyl acetate fraction of *Paeonia lactiflora* (EA-PL) presented the highest potency in scavenging DPPH radical when used in combination with four solvent-extracted fractions from AME (*P*<0.05), suggesting that EA-PL was rich in DPPH radical-scavenging activity (Fig.1A). Our results were in agreement with the previous report [19]. Herein, CIs were used to determine the possible interactive actions between the extracts or fractions. In order to calculate the CIs, dose-effect curves for the single extract or fraction were analyzed (data not shown). As shown in Fig.1B, the CIs for EA-PL+CF-AME and EA-PL+NB-AME were <1.0, indicating these combined extracts had a synergistic effect in scavenging DPPH radical. By contrast, the CIs for several combined fractions (i.e. CF-PL+EA-AME, CF-PL+NB-AME, and OH-PL+EA-AME) were >1.0, suggesting these combinations had antagonistic effects in scavenging DPPH radicals. As is well known, the phenolic and flavonoid compounds are the most commonly studied substances that greatly contributed to antioxidant activity of plant foods. Therefore, we measured the total phenolic and flavonoid contents in EA-PL+CF-AME combination, which exhibited the strongest activity in scavenging DPPH radicals. Results showed that the total phenolic and flavonoid contents were 603.339±22.894 mg GAE/g and 121.785±1.264 mg RE/g, respectively, which were significantly higher than those of other combinations (*P*<0.05, data not shown). These results suggested that the EA-PL fraction had the highest potency in scavenging DPPH radicals to warrant further fractionation. Thus, EA-PL was further chromatographed on a silica gel column (5.5×60 cm) using a stepwise elution of methanol/chloroform (methanol/chloroform  = 1∶19, 1.5∶18.5, 2∶18, 2.5∶17.5, 3∶17, 4∶16, 5∶15, 20∶0, v/v) to afford 8 fractions (A_1_-A_8_). Then, each fraction was combined with CF-AME to yield eight AME-PL combined extracts, i.e., E_1_ (A_1_+CF-AME), E_2_ (A_2_+CF-AME), E_3_ (A_3_+CF-AME), E_4_ (A_4_+CF-AME), E_5_ (A_5_+CF-AME), E_6_ (A_6_+CF-AME), E_7_ (A_7_+CF-AME) and E_8_ (A_8_+CF-AME). These samples were examined for *in vitro* antioxidant activity using DPPH free radical scavenging assay and FRAP test.

### *In Vitro* Antioxidant Activity of the Eight AME-PL Combined Extracts

The antioxidant activity might be attributed to different mechanisms, such as free radical scavenging, reducing potency, prevention of chain initiation, decomposition of peroxides and binding of transition metal ion catalysis [20]. Meanwhile, considering the complexity of the composition of herbal extracts, combined assays are needed for determination of their antioxidant activity [21]. Herein, both DPPH scavenging test and FRAP were applied for the evaluation of antioxidant activity of the AME-PL combined fractions.

#### DPPH Free Radical Scavenging Activity

**Figure 2:**
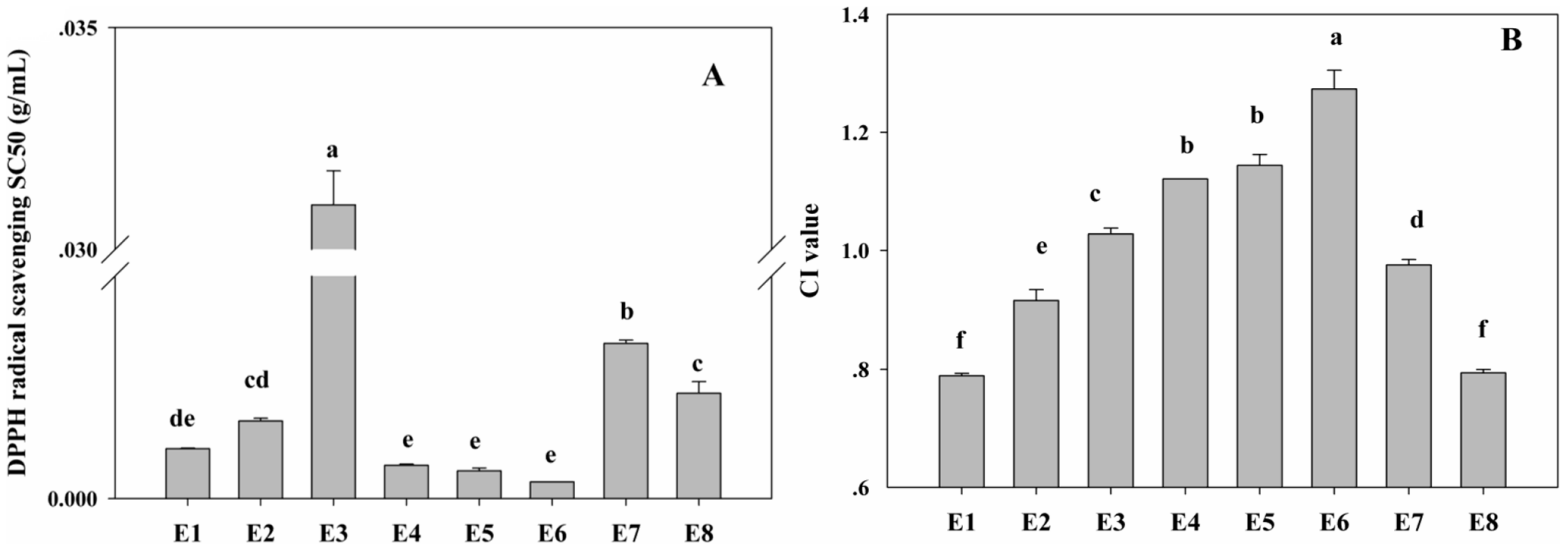
DPPH radical-scavenging activity (A) and CI values (B) of eight combinations of CF-AME and chromatographic fractions from EA-PL. Data are expressed as means ± SD (*n* = 3), and histograms marked with different letters are significantly different at *P*<0.05.

As shown in Fig.2A, the highest DPPH scavenging efficiency was observed in the groups treated with E_1_, E_4_, E_5_ and E_6_, and there was no significant difference between them. E_3_ exhibited the highest SC_50_ value of 0.031 g/mL, suggesting that it had the weakest ability in scavenging DPPH free radicals among the tested samples (*P*<0.05). The CI values for E_1_–E_8_ were calculated using CalcuSyn software and the results were illustrated in Fig.2B. It was found that E_1_ and E_8_ had the lowest CI values (CI = 0.789 and CI = 0.786, respectively), indicating they had the strongest synergistic effect (*P*<0.05). It is noteworthy that the combinations demonstrating the highest antioxidant effect did not show the lowest CI (e.g. EA-PL+CF-AME and EA-PL+EA-AME in Fig.1, and E6 in Fig.2). These findings demonstrated that the combination of two antioxidants may have strong antioxidant activity, but not necessarily engender synergistic efficacy, it may even generate antagonistic interaction (as indicated by their CIs>1.0). Previous literatures have showed that the synergism might arise from a complex interaction among single ingredients with different pharmacological functions, such that one ingredient enhances the therapeutic effect of another active ingredient [22], [23] or via coalistic combinations, such that all ingredients involved are inactive individually but become active in combinations [24]. It seems that the similar interactions among ingredients did not occur in the combinations tested in this study, and thus these combinations did not exhibit the antioxidant synergism.

#### Total Antioxidant Power Measured by FRAP Assay

**Figure 3:**
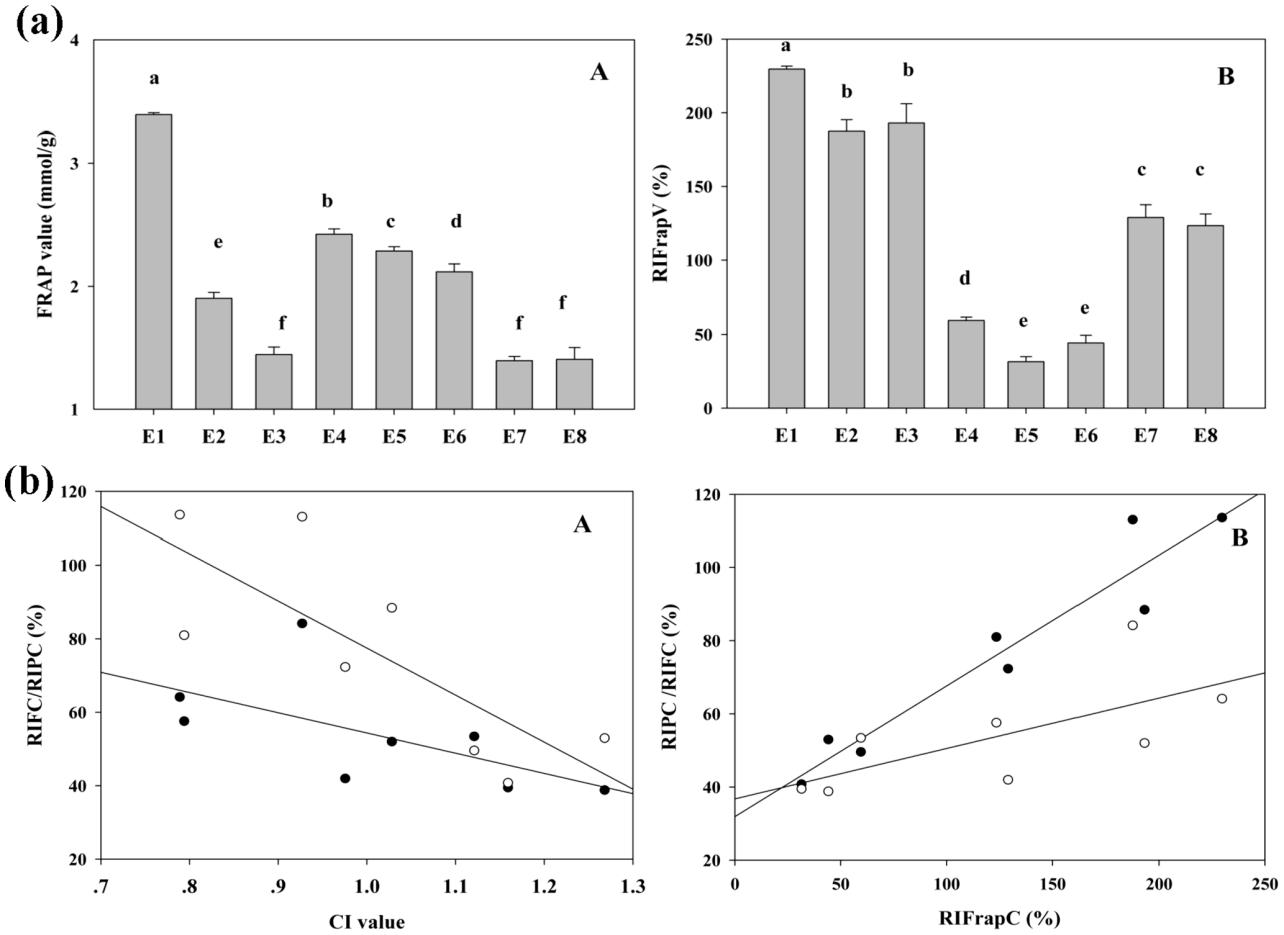
Ferric reducing antioxidant power of eight combinations of CF-AME and chromatographic fractions from EA-PL (a), and correlation between the rate of increase in total phenolic/flavonoid (RIFC/RIPC), CIs and RIFrapV values (b). (a-A) FRAP value; (a-B) RIFrapV is defined as the rate of increase in FRAP value; (b-A) Correlation between the RIFC/RIPC and CI values; (b-B) Correlation between RIFC/RIPC and RIFrapV values. Solid symbols represent the correlation between RIFC and CI or RIFrapV values, while open symbols represent the correlation between RIPC and CI or RIFrapV values.

The antioxidant activity of the eight combined extracts (E_1_–E_8_) was estimated by the FRAP assay, in which the antioxidants present in the sample reduce the Fe(III)/tripyridyltriazine (TPTZ) complex to the blue ferrous form, with an increase in absorbance at 593 nm. In order to obtain FRAP values, a calibration curve generated by analyzing standard FeSO_4_ solutions was derived from the absorbance versus concentration plot (*R* ^2^ = 0.9979). RIFrapV was also calculated as the rate of increase in FRAP value in the combined extracts as compared to theoretical sum of those of the respective herbs extract. As shown in Fig.3a-A, the highest FRAP value was observed for E_1_ (3.392±0.015 mmol Fe^2+^/g DW), suggesting that it had the strongest ferric reducing ability. The FRAP values for other samples decreased in the order: E_4_>E_5_>E_6_>E_2_>E_3_, E_7_, E_8_. As to RIFrapV, E_1_ exhibited a highest RIFrapV value of 229.68% (Fig.3a-B), showing that there was a synergistic enhancement in ferric reducing ability in E_1_. Additionally, the antioxidant activity of eight combined extracts, based on FRAP assay, was different from that based on the DPPH scavenging test. It might be explained by considering that these determinations were performed under different experimental conditions which were based on different reaction mechanisms. DPPH assay is believed to be one of the methods utilizing both hydrogen atom transfer and single electron transfer mechanism. The FRAP assay takes advantage of electron-transfer reaction and takes place at a faster rate than that for the DPPH test, whose degree of discoloration is attributed to the hydrogen donating ability of the test compounds [25].

### Total Phenolic and Flavonoids Contents

The contents of total phenolic and flavonoid has been proved to be positively correlated with antioxidant capacity [26]. Therefore, the variation in the contents of phenolic and flavonoid compounds in these combined extracts was investigated. RIPC and RIFC, defined as the rate of increase in phenolic and flavonoid contents in these eight combined extracts relative to the theoretical sum of those from the respective herbs extract, were calculated. Table 1 revealed that the total phenolic and flavonoid contents in all combined extracts exhibited various increases compared to their respective theoretical sum, as indicated by the RIPC and RIFC, respectively. The correlation between RIFC/RIPC and CI values as well as between RIFC/RIPC and RIFrapV value was further analyzed. As shown in Fig.3b, both RIPC and RIFC correlated significantly with the CI values, while a higher negative correlation was found between CI value and RIFC (*R*_CI-RIFC_ = −0.833, *P* = 0.010) than that between CI value and RIPC (*R*_CI-RIPC_ = −0.810, *P* = 0.015). Meanwhile, these was a positive correlation between the rate of increase in FRAP value and RIFC (*R*_RIFrapV-RIFC_ = 0.929, *P* = 0.001), significantly higher than that between RIFrapV value and RIPC (*R*_RIFrap-RIPC_ = 0.667, *P* = 0.05). These findings indicate that the variations in total flavonoids content might make a more significant contribution to synergistic antioxidant effect of the combined extracts. Our results were in agreement with the previous report that the antioxidant activity of eight traditional Chinese herbs pairs was determined or highly influenced by changes of their total flavonoid content [9].

**Table 1:** The contents of the phenolics/flavonoids and the rates of increase in phenolic/flavonoid contents in eight combined extracts from AME and PL.

	Total phenolic (mg GAE/g)^a^	RIPC^b^ (%)	Total flavonoids (mg RE/g)^a^	RIFC^c^ (%)
E_1_	144.572±1.100	113.643	89.568±1.169	64.171
E_2_	107.560±2.678	113.066	98.294±1.330	84.181
E_3_	75.426±2.743	88.421	73.975±1.981	52.030
E_4_	216.534±1.559	49.591	87.547±0.895	53.423
E_5_	254.471±3.346	40.801	78.280±3.301	39.491
E_6_	275.062±5.220	52.967	93.564±2.431	38.803
E_7_	97.007±1.707	72.348	64.549±1.323	41.969
E_8_	97.262±3.470	80.975	73.350±1.279	57.591

a Data were means ± standard deviation (n = 3).

b RIPC, the rate of increase in total phenolic content.

c RIFC, the rate of increase in total flavonoid content.

### Protection Against H_2_O_2_-induced Oxidative Damage in MRC-5 Cells

#### Cell Viability Assay

Since E_1_ exhibited a significant antioxidant synergism in both DPPH radical-scavenging and FRAP assays, we further examined its effect on H_2_O_2_-induced oxidative damage in MRC-5 cells. As shown in Table 2, treatment with 0.5 mM H_2_O_2_ led to a remarkable decrease in cell viability compared with control group (*P*<0.05). H_2_O_2_ plays a pivotal role among a great variety of ROS, and exogenous H_2_O_2_ can enter the cells and induce cytotoxicity due to its high membrane permeability damage in cells [27]. All the tested samples were able to significantly alleviate H_2_O_2_-induced oxidative damage in MRC-5 cells. Moreover, the groups treated with A_1_ and E_1_ had higher cell viability than that treated with V_E_ (*P*<0.05), indicating that they could provide stronger protection against H_2_O_2_-induced oxidative damage in MRC-5 cells. The cell viability for the tested samples and V_E_ was decreased in the order: A_1_>E_1_>V_E_>CF-AME. It is noteworthy that E_1_-treated cells have a significantly higher viability compared to the theoretical sum of those from A_1_ and CF-AME (*P*<0.05), suggesting a synergistic cytoprotection in the combination of A_1_ and CF-AME.

**Table 2:** The effect of different active extracts on cell viability, cellular antioxidant enzymes (Cu/Zn-SOD, Mn-SOD, GSH-Px and CAT) activities and MDA content in MRC-5 cells subjected to H_2_O_2_-induced oxidative stress.

	Cell viability (%)	Cu/Zn-SOD (U/mL)	Mn-SOD (U/mL)	GSH-Px (U/mL)	CAT (U/mL)	MDA (*µ*M)
Control	100.00±0.552^a^	16.138±0.074^a^	6.703±1.411^a^	17.143±1.276^a^	5.199±0.077^a^	1.667±0.112^f^
H_2_O_2_	57.247±0.288^g^	7.469±0.222^g^	0.999±0.428^d^	3.158±0.638^d^	0.248±0.032^e^	8.889±0.224^a^
V_E_+H_2_O_2_	68.726±0.521^e^	8.826±0.369^f^	4.706±0.652^bc^	9.925±1.276^c^	2.010±0.160^d^	5.316±0.112^c^
CF-AME+H_2_O_2_	67.058±0.185^f^	9.558±2.221^e^	5.014±0.521^b^	5.414±1.276^d^	1.852±0.064^d^	7.222±0.114^b^
A_1_+H_2_O_2_	83.509±0.236^b^	12.691±0.074^b^	3.259±0.019^c^	13.083±0.638^b^	3.568±0.064^b^	3.730±0.112^e^
E_1_+H_2_O_2_	77.669±0.352^c^	12.012±0.148^c^	4.998±0.316^b^	10.827±1.276^bc^	3.500±0.032^b^	4.841±0.337^d^
TS#(A_1_+CF-AME+H_2_O_2_)	75.283±0.173^d^	11.124±0.148^d^	4.136±0.270^bc^	9.248±0.318^c^	2.710±0.001^c^	5.476±0.116^c^

Data were means ± standard deviation (n = 3).

a-g Different letters within the same column indicated that means were statistically different at *P*<0.05.

# TS: the theoretical sum of the antioxidant enzyme activity or MDA content of respective constituent extract with their proportion being 1:1 in the combinations.

#### SOD, CAT, GSH-Px Activities and MDA Content

Oxidative stress induced by H_2_O_2_ resulted in lipid peroxidation and destruction of cell membranes, producing secondary products such as the MDA, and thus damaged the integrity of membrane and/or membrane-associated function in subcellular organelles such as mitochondria, microsomes and lysosomes [28]. The damages induced by ROS are also associated with the inactivation of endogenous antioxidant enzymes, such as SOD, CAT and GSH-Px, which are important for evaluating the effects of free radical scavenging activity and guarding against superoxide toxicity [29]. In order to gain some insights into the synergistic mechanism of AME and PL, we further investigated the effect of E_1_, A_1_ and CF-AME on the antioxidant enzymes activities (SOD, CAT and GSH-Px) and MDA content in H_2_O_2_-induced MRC-5 cells.

As shown in Table 2, in the normal MRC-5 cells, the activities for Cu/Zn-SOD, Mn-SOD, GSH-Px and CAT were 16.14±0.07 U/mL, 6.70±1.41 U/mL, 17.14±1.28 U/mL and 5.20±0.08 U/mL, respectively. Upon the treatment with 0.5 mM H_2_O_2_, their activity were decreased to 7.47±0.22 U/mL, 1.00±0.43 U/mL, 3.16±0.64 U/mL and 0.25±0.03 U/mL (*P*<0.05), respectively. Supplementation of E_1_, A_1_, CF-AME and V_E_ significantly inhibited the loss of these antioxidant enzymes activities in H_2_O_2_-stimulated cells (*P*<0.05). A_1_ exhibited the extremely high efficiency in protecting the activities of Cu/Zn-SOD, GSH-Px and CAT enzymes. The highest Cu/Zn-SOD activity was observed in the cells treated with E_1_. Moreover, E_1_ showed comparable potency to A_1_ in elevating the activities of GSH-Px and CAT. The accumulation of MDA is a sensitive indicator of the peroxidation of cellular lipids in cultured cells. The measurement of MDA in H_2_O_2_-induced MRC-5 cells provides an alternative to evaluate the protection by the herbs extracts. Stimulation of MRC-5 cells with 0.5 mM H_2_O_2_ led to a dramatic increase in MDA content from the basal level of 1.67±0.11 *µ*M to 8.89±0.22 *µ*M. All the tested samples significantly attenuated the H_2_O_2_-elicited increase in MDA level (*P*<0.05), of which the strongest effect was observed in the group treated with A_1_, followed by E_1_ and V_E_. It is noteworthy that E_1_ could protect the activities of almost all antioxidant enzymes and decrease MDA content better than the theoretical sum of A_1_ and CF-AME, although no significant difference was observed in the activities of Mn-SOD and GSH-Px between E_1_ and theoretical sum of the respective constituent.

### Identification of Potential Antioxidant Components in E_1_

**Figure 4:**
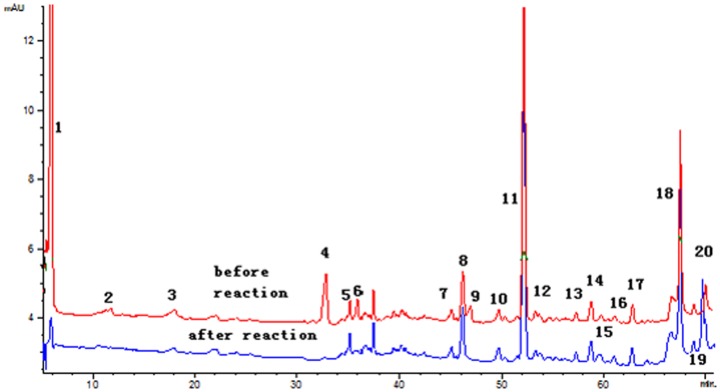
HPLC-DAD chromatograms of E_1_ before and after reaction with DPPH radicals.

The on-line DPPH-HPLC method has been widely used for screening of the potential radical-scavenging components from various food extracts [30]. After reaction with DPPH, the peak areas (PAs) of the radical scavenging compounds would obviously disappear or decrease in UV chromatograph, while for those without antioxidant activity, there was almost no change in their PAs. Herein, this approach was employed to screen the potential antioxidant compounds from E_1_. HPLC profiles of E_1_ with or without DPPH treatment are illustrated in Fig. 4. It was found that eight peaks (1, 3, 4, 6, 7, 9, 10 and 20) were decreased in the PA after spiking with the DPPH free radicals, suggesting these components could be regarded as the potential antioxidant candidates of E_1_. Among the identified peaks, peaks 1, 3 and 4 were present in PL, while the others (6, 7, 9, 10 and 20) were from AME. Further characterization of these potential antioxidant components was carried out by HPLC-MS/MS, owing to it can provide affluent multistage fragment information for compounds with collision-induced dissociation [31]. As shown in Table 3, seven antioxidant components were unambiguously identified as Oxypaeoniflora (1), Catechin (3), Calycosin-7-O-β-D-glucopyranoside (6), Fomononetin-7-O-β-D-glucopyranoside (7), 9,10-dimethoxy-pterocarpan-3-O-β-D-glucopyranoside (9), 2′-dihydroxy-3′,4′-dimethyl–isoflavan-7-O-β-D-glucopyranoside (10), Quercetin (20), by comparing their UV maximum absorption wavelength, MS and MS/MS data with those of the standard references, as well as the previous reports [32], [33]. It is not surprising that most of these components belonged to the flavonoids family, which has been confirmed to be excellent antioxidants.

**Table 3:** Data from HPLC-DAD-ESI-MS/MS for characterization of potential antioxidant components in E_1_.

Peak^a^	*t*_R_ a (min)	[M+Na]^+^ (m/z)	[M+H]^+^ (m/z)	[2M+Na]^+^ (m/z)	MS/MS (m/z)	UV λ_max_ (nm)	Assigned identity
1	6.334	-b	497	-	477, 335	254	Oxypaeoniflora
3	17.047	-	291	-		280	Catechin
6	34.198	-	447	915	285	225, 248, 282	Calycosin-7-O-β-D-glucopyranoside
7	45.616	-	431	-	269	250, 258	Fomononetin-7- O-β-D-glucopyranoside
9	49.212	485	-	-	301	229, 284, 344	9,10-dimethoxy-pterocarpan-3-O-β-D-glycoside
10	50.967	487	-	-	482, 303	280	2′-dihydroxy-3′,4′-dimethyl-isoflavan-7-O-β-D-glucopyranoside
20	69.749	-	303	-	285, 257, 167	254, 370	Quercetin

a Peak numbers and retentions time refer to Fig.4.

b -, not detected.

In the present study, we found that the total phenolic and flavonoid contents in combined AME and PL extracts varied differently from the theoretical sums of those from the respective herbs. A significant correlation was also observed between the increments of total phenolic/flavonoid and the elevation of antioxidant capacities in these combinations. On-line DPPH-HPLC-MS/MS analysis led to the identification of seven potential antioxidant compounds. These results suggested that the changes in the content of total phenolic and flavonoid, especially the flavonoid, might contributed greatly to the antioxidant synergism in combined AME and PL extracts.

## Conclusions

Eight AME-PL combined extracts (E_1_-E_8_) were prepared based on bioactivity-guided fractionation, and their *in vitro* antioxidant activity was examined. E_1_ demonstrated the strongest synergistic effect in scavenging DPPH radicals and reducing ferric ions (*P*<0.05). Moreover, E_1_ also presented strong cytoprotection against H_2_O_2_-induced oxidative damage in MRC-5 cells by restoring the cellular antioxidant enzymes activities. Finally, seven antioxidant substances were identified in E_1_ as oxypaeoniflora, catechin, quercetin, calycosin-7-O-β-D-glucopyranoside, fomononetin-7-O-β-D-glucopyranoside, 9,10-dimethoxy-pterocarpan-3-O-β-D-glucopyranoside, and 2′-dihydroxy-3′,4′-dimethyl–isoflavan-7-O-β-D-glucopyranoside. These findings may provide some basis for the purported synergistic effects of traditional Chinese herbs, and facilitate their utilization in combination as functional foods and dietary supplements.
